# Up against the System: A Case Study of Young Adult Perspectives Transitioning from Pediatric Palliative Care

**DOI:** 10.1155/2013/286751

**Published:** 2013-08-12

**Authors:** Karen Cook, Harold Siden, Susan Jack, Lehana Thabane, Gina Browne

**Affiliations:** ^1^School of Nursing, McMaster University, Faculty of Health Sciences, c/o Dr. Gina Browne, Health and Social Service Utilization Research Unit, McMaster Innovation Park, 175 Longwood Road South, Suite 210A, Hamilton, ON, Canada L8P 0A1; ^2^Department of Pediatrics, University of British Columbia, Room F602A, 4500 Oak Street, Vancouver, BC, Canada V6H 3N1; ^3^School of Nursing, McMaster University, Faculty of Health Sciences, Health Sciences Centre, Room 2J32, 1280 Main Street West, Hamilton, ON, Canada L8S 4K1; ^4^Department of Clinical Epidemiology, Centre of Evaluation of Medicines, 1050 Main Street East, Level P1, Hamilton, ON, Canada L8N 1G6; ^5^School of Nursing, Health and Social Service Utilization Research Unit, McMaster Innovation Park, 175 Longwood Road South, Suite 210A, Hamilton, ON, Canada L8P 0A1

## Abstract

Advances in pediatric care have not provided the interdisciplinary support services required by those young adults with pediatric life-threatening conditions (pedLTCs) who live beyond childhood but have limited expectations to live past early adulthood. These young adults, the first generation to live into adulthood, face multiple challenges transitioning from a plethora of pediatric palliative services to scant adult health services. In a case study, using an innovative bulletin board focus group, we describe the complex interplay of the health, education, and social service sectors in this transition. Our descriptions include system deficits and strengths and the young adults' resilience and coping strategies to overcome those deficits and move forward with their lives. Young adults with pedLTC need knowledgeable providers, coordinated and accessible services, being respected and valued, and services and supports that promote independence. We recommend implementation of multidisciplinary solutions that are focused on young adult priorities to ensure seamless access to resources to support these young adults' health, educational, vocational, and social goals. The input and voice of young adults in the development of these services are imperative to ensure that multisystem services support their needs and life goals.

## 1. Introduction

A life-threatening condition adds complexity to the challenging period between childhood and adulthood and the needs and experiences of young adults are specific and different from children's and adults needs [1]. Unfortunately, advances in pediatric care have not provided the interdisciplinary support services required by those young adults with pediatric life-threatening conditions (pedLTC) who live beyond childhood but have limited expectations to live past early adulthood [2–4]. These young adults, who are the first generation with pedLTC to live into adulthood, face a multitude of challenges transitioning from the plethora of pediatric services to scant adult health services [5] and are vulnerable to a significant deterioration in health status and even increased mortality once they leave pediatric care [3, 6]. While the number of young adults with pedLTC is small, the numbers continue to rise [7]. In the United Kingdom, the number of 16–19-year olds living with life-threatening conditions nearly doubled in a decade [1]. To date, there is a paucity of research about the transition experiences of youth “aging out” of pediatric palliative care [3].

Palliative care is an interdisciplinary approach to the management of incurable or life-threatening diseases [8]. However, the philosophical differences between pediatric and adult palliative care exclude this population from adult palliative care services because pediatric palliative care is lifelong until death, whereas adult palliative care focuses on the last weeks and months of life [7, 9]. The differences in definition and types of services offered in palliative services for youth and adults have resulted in confusion and abrupt ending of supportive services for young adults with pedLTC [10]. Currently, young adults with pedLTC live in a zone of disability and chronic illness that has no specific care delivery system [11]. 

### 1.1. Background: The Transition Experience

While not specific to young adults with pedLTC, the transition literature for youth with complex conditions does provide insights about this complex phenomenon. Transition is the planned and purposeful movement of youth with chronic medical conditions from comprehensive pediatric services to adult-oriented health care [12]. Preparatory transition work occurs between 12 and 18 years of age, but the real lived experience of the transition occurs in the early adult years [13, 14]. Many comparisons have been discussed in the chronic health transition literature between pediatric and adult healthcare systems, which also apply to the experiences of young adults with pedLTC. In the pediatric system, the health care team and parents make decisions with or in the best interest of young people. In adult care, young adults are expected to navigate new systems and make their own decisions [15–17]. This approach does not consider the impact of the young adult's cognitive and verbal abilities, patterns of family decision making, adjustments to their condition, and other factors that may leave the young adult vulnerable in the adult system.

The experience of navigating the transition process is influenced by multiple and diverse factors [3, 18] such as the skills and knowledge of the youth and their families [19], the training, expertise, and coordination of health care providers and services in both the pediatric and adult sectors [20], family support [21, 22], and the availability of education and social service system supports [3, 23]. To date, research in transition has focused on specific health conditions, such as diabetes, cystic fibrosis, and congenital heart defects [2, 13], and on specific skill acquisition for youth and pediatric, rather than adult health care providers [3]. Further, the approach to care for young adults becomes medical specialty focused (respirology, cardiology, etc.), rather than attentive to whole person care, which is essential for symptom management, care planning, and quality of life decision making. 

However, social and educational supports are equally important in the transition phase. A community-based approach integrating health and social services that include developmental components of young adulthood such as independent living, social, educational, and career needs is recommended [24–26]. In addition to improved services in adult care to accommodate their changing health status and learning to navigate new systems, support for the enormous psychological issues, such as the impact of their condition on themselves and their family, is also recommended [5]. 

For young adults with pedLTC, opportunities to practice taking on the responsibility of their own health care can be limited because of repeated and prolonged hospital stays, intrusive home care regimens, and dependence on others for care, learning disabilities, sporadic school attendance, and fewer socialization opportunities [5, 27, 28]. In addition, for young adults who are cognitively capable, the continuation of family centered care through adolescence limits their opportunities to develop autonomous health self-management skills [11]. For young adults with pedLTC, learning to navigate adult systems early in the transition process is important.

Finally, pedLTCs are rare and unfamiliar in adult care. This has resulted in a dearth of experienced adult clinicians and interdisciplinary care groups that understand and have the resources to manage this group's complex care needs [12, 29]. Adult general and specialist health care practitioners may be unwilling to accept a youth with pedLTC into their care because of the time required to manage the complexities of the youth's condition, as well as their own unfamiliarity and lack of expertise with these rare pediatric conditions [13, 30–32]. Primary care providers might have only one pedLTC patient in their entire career and adult specialists are not trained in pediatric conditions [30, 33]. Therefore, for young adults with pedLTC there is no real system to accept them after pediatric care [11]. 

## 2. Methods

### 2.1. Research Design

This paper is part of a larger descriptive case study, guided by Yin [34], that explored (1) the experiences of young adults with pedLTC who have transitioned from pediatric palliative services to adult care, the significant supports and factors that contributed to or were barriers to achieving their developmental goals and (2) the advantages and disadvantages of an online focus group to engage medically fragile participants. Propositions guide case study data collection and provide boundaries for analysis. The propositions for “Up Against the System” are listed in Table 1.

The objective of this paper is to describe the complex interplay of the health, education, and social service sectors in the transition experiences of these young adults. Accessing this population with traditional qualitative interview methods, such as focus groups, is difficult because their numbers are small; they are geographically dispersed; the complexity of their conditions requires many supports such as care attendants for personal care and respiratory support, equipment such as power wheel chairs and computer-assisted communication devices; and they have divergent mobility and communication abilities related to the impact of their conditions. An innovative method of data collection to overcome these barriers is described below. Two university ethical review boards approved this study.

This case study was conducted in two phases. The first phase consisted of three in-depth face-to-face interviews with two participants. Its goals were to understand their transition experiences with health, social, and educational services, and to compare their experiences with the research literature and practice recommendations on transition for youth with chronic health conditions. The second phase of this case study included the development, testing, and implementation of a bulletin board focus group (BBFG), which is an asynchronous modification of an online focus group. Content for the BBFG discussion was developed utilizing the intensive one-to-one interviews described above, in addition to consultations with a pediatric hospice transition team, a young adult with a pedLTC who exceeded the age criteria for this study, and pedLTC experts in health and social services.

### 2.2. The Sample

A purposeful sample of youth with pedLTC, aged between 19 and 29 years, with limited expectations to live beyond their first decade of adulthood (29 years), having pedLTC such as Duchennes', Muscular Dystrophy, Spinal Muscular Atrophy, Friedrichs Ataxia, and undiagnosed conditions, was selected from the graduates of a children's hospice in Western Canada. Within the hospice, the numbers of youth with pedLTC transitioning from pediatric hospice are very small (8–10 per year), and about half of these graduates met the following selection criteria: English speaking, cognitively able to participate in their care and decision making, owning a computer, and having either verbal or typing ability sufficient to participate in face-to-face interviews or an online focus group. The hospice has transitioned approximately 97 youths from its program in the past 15 years. Of these, approximately half were eligible for this study. Most of these young adults require 24-hour attendants for personal and respiratory and feeding support. Their independent function may be limited to minimal movement of their index finger or a dot on their forehead to control their power wheel chairs, computers, and phones. All the participants have lived with their conditions for more than 10 years, and most were diagnosed when they were less than 5 years old. 

For ease of reading and protecting confidentiality, the young adult participants are referred to as “he” and all other participants as “she.”

### 2.3. Data Collection

Data for this two-phase study was collected over 12 months. In the first stage of this case study, two young adults with diverse and unique experiences that differed regarding characteristics, such as diagnosis, age, gender, family support, opportunities for postsecondary education, and living arrangements, were invited to participate in three face-to-face interviews. These participants were similar in their physical limitations requiring 24-hour care and possessing minimal movement adequate to control their power wheel chairs, computers, and phones. One participant was able to speak directly with me, and the other used a computer-assisted device to type out his answers.

In the second phase of this study, a BBFG was conducted with two groups of four young adults with pedLTC. Goals were to further explore the key issues and experiences described by the face-to-face participants, extend understanding of their range of experiences and successes and barriers within the health, education, and social sectors, and provide recommendations for change from the perspectives of young adults. These participants were selected to achieve variety among their diagnoses, gender, age, and posttransition experiences. The BBFG provided an opportunity to bring together small groups of young adults with pedLTC, who otherwise could not be convened. This is the first circumstance of using a BBFG to gather data from this population. 

Each BBFG was conducted for five days. Daily discussions were set up by the researcher/moderator using a combination of text- and video-based questions. Participants logged in and out of the discussion at their convenience answering questions posted on the discussion board, follow-up questions posted by the moderator, and those posted to each other in response to other participants' comments. Participants chose text or video options to respond to the discussion questions. The discussion became interactive as the participants responded to the moderator's questions and each other's responses. The greatest strengths of the BBFG were (1) the appeal of this methodology for young adults, (2) creating a forum discussion for young adults with pedLTC that would not have been possible due to the logistical difficulties associated with their conditions, variant communication modes and barriers, and financial costs, and (3) in addition to its value as a research method, the multiday focus group became a community for the participants. Participation rate was very high with respondents answering an average of 96% of the questions and contributing to the discussion every day.

### 2.4. Data Analysis

Face-to-face interviews with participants and experts in pedLTC were taped, transcribed, and imported into the NVivo 9 software analysis program. Transcripts from the focus groups that included all of the contextual content of the conversations such as capitalization, multiple exclamation marks, emoticons, and photos were also imported directly from the iTracks discussion board into NVivo 9. Conventional and directive content analyses [35, 36] were used along with a constant comparative analytical process to review emerging patterns, themes, variations, and relationships among the data sets [37, 38]. Case study analysis was directed and confined by the theoretical propositions that frame the study [34]. 

Credibility was ensured through use of complementary case study data collection strategies, such as the face-to-face interviews, online focus groups, and content analysis of the literature, program recommendations, and professional and jurisdictional policies. These varied strategies triangulated the data sources, prolonged engagement with participants, documents, and data, and facilitated persistent observations, member checking, and searching for disconfirming evidence [39–42]. Specifically, the BBFG strengthened credibility through (1) high response and retention rates, indicating that participants were interested and engaged over a long period of time [40, 41], (2) the immediate generation of transcripts directly from the participants' responses, including every word and textual descriptor [43], and (3) a constant member checking, as participants' postresponses to both the researcher and other participants. Finally, a pediatric palliative program transition expert and a physician and two participants from the BBFGs reviewed the results for credibility.

## 3. Results

### 3.1. Characteristics of the Participants

Fourteen participants were recruited to participate in this study. Two participated in face-to-face interviews. Of the remaining 12 participants, 8 agreed to participate in the BBFG. Refer to Table 2.

### 3.2. Up against the Systems

The Canadian Pediatric Society [44] stresses that transition goals should include the adolescent's realization of personal potential for activity, education, recreation, and vocation, completion of adolescent developmental tasks, and the attainment of self-esteem and confidence. Ideally, young adults with special needs can achieve a self-directed life. However, when faced with the inevitable gaps in the systems and lack of coordinated services among the health, educational, and social sectors, the participants employed various thoughtful and novel strategies to get what they needed to move forward with their lives. When they came to an impasse in the education and social support systems, they found more opportunities to navigate around or negotiate for what they needed than when they encountered barriers in the health care system. Their successful strategies included thorough research to determine all the available services and how to access them; using their “village” for ideas (talk to others who have succeeded ahead of you) and advocacy (asking influential people, such as civic and health care leaders, to write a support letter); being persistent, positive, and creative; and never taking no for an answer. In some circumstances, the systems were too formidable to impact, and they were stalled or unable to move forward. 

The following section describes what the young adults in this study most want from the health, education, and social systems: knowledgeable providers, coordinated and accessible services, being respected and valued, and services and supports that promote their independence. System deficits and strengths and the young adults' resilience and coping strategies to overcome the deficits to get what they need to move forward with their lives are also described.

#### 3.2.1. Knowledgeable Providers

Participants had varying levels of satisfaction with their health care providers. The participants referred mostly to their physicians because they did not have access to multidisciplinary health team members. For this population, access to individual adult specialists who had comprehensive understandings of their condition was difficult; access to a team of physician experts was impossible. Many found that their physician's knowledge about their pedLTC was limited, and they were educating their doctor about their condition. As one of the young adults explains, *“it's been really challenging for me because not only do I have complex health issues, but I have to educate my doctors about what (my condition) is.” *Further, even adult specialist palliative care was not a supportive experience because of lack of expertise in pedLTC and young adult developmental goals. 

Without understanding of these pedLTCs, health care providers were unable to provide anticipatory guidance and management strategies. Reactive rather than proactive care was one of the most significant changes and struggles described by these young adults. One participant commented:

*When I was a kid it was like the doctors kept on top of my medical condition and everything involving it. In adulthood I really have to seek out any sort of help when it comes to specialists and doctors.*

Compared to their solo adult practitioners, their multidisciplinary pediatric teams were perceived as “very active” with information beyond health care needs that included housing options, equipment, and support for education. When condition specific expert care was not available, the young adults remained in acute care settings for extended periods of time or received standardized medication protocols for the “normal” population that resulted in untoward side effects and consequences for young adults with pedLTC.

Knowledgeable care was also compromised by ineffective communication among care providers. This added stress for the youth, or in some cases, family members or their pediatric health team, who had to advocate, update, and educate each of their new adult health care providers. Some young adults were unable to take on this responsibility and bridge the communication gap among their care providers because of the complexity of their condition, resulting in declining communication abilities, and access barriers such as re-referrals.

When their conditions were stable, participants were satisfied with the knowledge of their health team and access to the care and services they received. They were empowered by the experience of taking control and being their own advocates. These participants had not experienced any recent changes in their conditions and while they were stable, they did not *“need a team hovering over me all the time.”* However, they were aware that, for their friends whose health was not stable, access to care was difficult and not easily coordinated. While all participants acknowledged that their health status would decline, they were overwhelmingly hopeful and positive that their conditions would remain stable for the next 5–10 years. Anticipating a decline in their condition in the future, they indicated that they would prefer an expert team of health care providers, similar to their pediatric palliative care experiences, including specialist nurses. Only a few participants had experience watching a friend die with their same condition, and most participants had not planned for their declining health, preferring to focus on their current stable condition and hopeful that it would remain stable or that new technologies and cures would be discovered before they faced declining function and symptoms. 

#### 3.2.2. Coordinated and Accessible Services

During their pediatric health care, efforts had been made to coordinate appointments with their multiple care providers. This was facilitated by the proximity of physicians and multidisciplinary team members within the same hospital. For young adults with neuromuscular type pedLTC in the adult system, the organization of separate appointments with their primary care physician, specialists in respirology, neurology, cardiology, gastroenterology, physical, and occupational therapy, and nutrition, medical and oxygen suppliers becomes daunting. Getting to doctors' and service providers' offices is a Herculean effort, requiring special transportation accommodating their power wheel chairs, organization of their supplies and equipment, and availability of parents, partners, or care aides to attend the appointments with them. Required repeat visits to their family physician to access their specialists added even more appointments and logistical problems, as described by this participant:

*It can be really annoying because like every six months if you don't go see your specialist then they close your file and you have to get a new referral each time. And for a complex... disease that will go on for my whole life, like it's not a temporary thing.... *

Accessibility to health services was improved when there was prompt coordination of care among their physicians. Participants who were satisfied with their access to health services described a coordinated effort between their primary care provider and adult specialists, which resulted in quick and efficient response to symptoms and subsequent treatment in hospital. 

Reliable and prompt access to health care services can be problematic, especially for those young adults living independently or in extended care facilities. For example, when a participant's call bell was not answered and he required suctioning, he “face booked” his friends and asked them to call the nurse's desk at the extended care facility to tell them that he needed help right away. In another example, a participant was unable to travel by ambulance to hospital because lying down was too painful. He was transported to the hospital emergency room in a police “drunk tank van” that accommodated his power wheelchair and returned to his facility via an out-of-service city bus. While these examples are unusual, they do demonstrate the significant gaps in services and safety that arise for young adults with pedLTC who do not have a dependable network of support.

 Problems with access to academic and social support programs were universal. Participants emphasized the need for an integrated program that provides seamless resources and supports between these two systems. For example, for those attending postsecondary education prior to turning 19, many experienced a one-year gap between the end of funding from the pediatric program and the start of adult funding, resulting in inadequate resources for both home and school support. 

In addition to coordination gaps between the systems, accessibility to postsecondary education was hindered by their high schools' and postsecondary counselors' lack of knowledge about the grants, programs, and care aid funding available to support their education. For the participants attending postsecondary institutions, accessing the resources they needed required lobbying the student assistance departments that were intended to support them with funding and accommodations. Asking influential pediatric health care providers to write a letter on their behalf was one successful strategy that secured necessary support. For example, one participant who fought to have bathroom accommodations in place so that he could attend university said *“I think that my advisor decided to change her mind because there were some people advocating with me and she saw that there was a need, and if she didn't install an accessible washroom for me it would be considered discrimination.” *If lobbying efforts were not successful, or if the participants did not have personal resources to pay for tuition, accommodative renovations to postsecondary facilities, and attendant's wages, education plans were not achievable or were postponed. Even while their conditions were stable, fatigue, sickness, and illness and communication restrictions impacted their ability to achieve their postsecondary goals. Further delays to secure the necessary supports could mean shortening or preventing the pursuit of their educational dreams as their health condition further changes and deteriorates. 

Only one participant who attended a postsecondary institution described receiving sufficient support, resources, and guidance. 

#### 3.2.3. Being Respected and Valued

In addition to improving the delivery of services in the health, education, and social systems, being respected by the providers of these services was also highly valued. For example, health services focused on disability rather than ability were viewed as condescending:

*The doctors who specialize in “normal” things sometimes look at me like I have a disability and I deserve to be treated differently because of that. They've not been very understanding that we all just want to feel and live as normal a life as everyone else.*

While young adults with pedLTC are disabled, normalization in their lives is important, including interactions with their health providers. 

The participants attending postsecondary education experienced being devalued as potential students and being questioned about their cognitive capabilities because of their disabilities. One participant was told that his* “needs were too intensive”* and many of the students related that when they asked their professors questions about their class, the professor spoke to their care attendant instead of them. Patronizing and condescending attitudes that felt demeaning and inconsistent and incorrect information about resources and services for which they were eligible limited their access to and funding for postsecondary education. 

#### 3.2.4. Opportunities for Independence

As the first generation of young adults with pedLTC to live into adulthood, these young adults are pushing the current system boundaries to get the supports and resources they need for independent living. Their paradox is that gaining independence necessitates being dependent. Their community of caregivers, family, and friends and their equipment and supplies are their means to independence. As one of the participant describes, “*there are some things I need assistance with, and even though I'm super stubborn, these are things I just have to accept.”* For those with a tracheostomy, an attendant is necessary 24 hours a day. However, for some, short periods of being alone are worth the risk of the tracheostomy becoming dislodged and suffocating without help. Recognition and acceptance of their dependence on physical support in no way diminished their strong resolve for independence. 

Of all the system-focused struggles identified by these young adults, achieving sufficient support for independent living was the most difficult. Acquiring sufficient attendant time and resources from the program designed to support their independent living required sophisticated research and advocacy skills. While all of the participants hoped to have an opportunity to live independently, efforts to procure funding support for care attendants and equipment such as specialized beds and computer-assisted technology for safety, plus the cost of renting accommodations suitable for their power wheel chairs and other equipment, was not only frustrating but in most cases insurmountable. One of the 10 participants was living independently during data collection, partly made possible by having a spouse. Another participant was in the process of lobbying for support to move from an extended care facility to his own apartment. Thirty-five care providers and managers from the health and social service sectors attended a meeting to discuss resource allocation and coordination for one person's move. Equally frustrating for participants were either experiencing “no expert availability” or dealing with too many experts who did not coordinate their roles and services. 

Some young adults with pedLTC live in extended care facilities because they do not have the support to live at home or independently. Here, it was found that young adult developmental goals and desire for independence clashed with the culture of extended care. Even for those who lived by a mantra of *“never give up fighting,”* opportunities to achieve their personal goals were constrained and deflated by maintenance style care that did not encourage or support their independence or education, recreational, and vocational goals. For those who are not “fighters,” hopefulness became hopelessness. 

### 3.3. Making the Systems Better

Young adults with pedLTC, having grown up in the pediatric health care system and experienced the challenges of transition and adapting to an adult health care system that was not designed nor equipped to meet their needs, can offer invaluable perspective to practice and policy leaders regarding how to improve the systems.

The young adults in this study recommended a team approach to treating their medical condition, an extended transition period over five years that facilitated consultation between their pediatric and new adult providers, access to a multidisciplinary clinic of experts related to their conditions, one-time referrals to specialists that would not require repeat visits to a family physician, and more communication between their family physician and adult specialists to keep their family physician apprised of their care needs. They also hoped for medical and technological advances that would continue to improve their health status. 

Desired improvements to the education system included more funding support for tuition and their personal care attendants. A forum in which people transitioning with disabilities could learn about the resources available at each postsecondary institution how to access these services, and how to lobby for supports not currently available were also recommended.

In the social services sector, their suggestions included changing the age at which adult funding started to coordinate with the end of pediatric funding, and increasing eligible hours of care to support independent living. One participant recommended creation of a service that he would call “HandicApparel,” from which medical equipment could be accessed for reasonable prices. Access to a place to get away (similar to pediatric hospice) with support for their personal care, end of life care, and opportunities to socialize with other young adults with similar conditions were also proposed.

## 4. Discussion

While there has been extensive research, description, and opinion reported on transition challenges and solutions for youth with chronic health conditions, this study is among the first to describe the unique transition experiences for young adults with pedLTC. The participant data exemplifies the multiple intersecting factors that influence the transition experiences of young adults with pedLTC: their individual characteristics, such as skills and knowledge for self- or directed care; interpersonal support from their family, friends, and community; organizational coordination and support between pediatric and adult services; and policy resources to support transition [3]. Further, the participant responses demonstrate the creative and time-consuming work the young adults, their families, volunteers, and professionals have undertaken to overcome system barriers and initiate changes that need to be normalized across all the health, education, and social service sectors. 

### 4.1. System Improvements Are Necessary

The current health, education, and social systems do not fully support the transition of youth from pediatric to adult services. The complexity of the medical transition process has resulted in a fragmented focus on health care transitions that mostly exclude the social and educational systems [3, 23, 25]. However, even if well integrated, health care services do not create a healthy community [25]. For example, education is one of the three strongest determinants of health and wellbeing for adolescents and young adults [45], but the young adults in this study faced barriers and prejudice accessing postsecondary education. Successful transitions require policy approaches that move beyond health care needs and recognize the full range of young adult developmental needs [46]. Cross-system linkages are necessary to facilitate timely and efficient support between the pediatric and adult health, education, and social systems to support and enhance these young adults' limited remaining years. Most importantly, modifications to current practices and development of new services and programs should be informed by the views of the young people themselves.

From a systems perspective, there is not an equivalent system or constellation of coordinated services for young adults with pedLTC to transition into after pediatric services [3]. Adult health, education, and social systems do not coordinate or integrate their services and do not have central leadership, resulting in mismatched programs with differing intentions. Health care system transition includes the interacting stakeholders (young adults, family members, health care providers, administrators, and policy makers); pediatric and adult health care providers and organizations; and the specific transition interventions [3]. 

Bridging the chasm between pediatric and adult health care is especially complex for young adults with pedLTC. First, prior recommendations for improving transition from pediatric care to adult care such as (1) starting preparation for transition in the early teen years, (2) specific guidelines to ensure that requisite skills and knowledge are achieved prior to transfer, (3) training of adult health care providers, and (4) ensuring efficient transfer of comprehensive health and personal information [12, 26, 47] remain unattained for young adults with pedLTC and their providers. Further, many of these young adults receive all of their health care through their pediatrician. When they reach adulthood, their adult primary care provider is not familiar with their health needs, coordination of services, and preferences. Ensuring that youth are seen by their primary care providers throughout their specialized pediatric care and improving interconnectedness between pediatric and adult services may create accountability for young adults dropping out of adult care and accelerate adult services to adapt to the needs of young adult patients [48].

Second, symptom management for young adults with pedLTC becomes more complicated in adult health care because of the broad array of rare and unknown conditions and few corresponding opportunities for adult health care providers to acquire specialized knowledge. Without access to supportive health services, young adults lose hope and power waiting months to see an adult specialist for symptom relief. Moreover, pedLTCs in young adults often follow a series of declining-plateaus in health status that are punctuated by periodic life-threatening symptoms with little predictability about which crisis event will be life ending [49–52]. With limited and unknown time remaining in their lives, waiting months for symptom relief has a significant and detrimental impact on the quality of their life.

 Third, previous research has found that adolescents with chronic illnesses were less likely to want to discuss their end of life preferences than those who were healthy [53]. Therefore, expert knowledge about symptom management and end of life trajectory, a trusting relationship with adult care providers skilled in end of life planning and a coordinated plan for medical emergencies are essential components of health system support for young adults with pedLTC [54].

Fourth, better access to coordinated physician services will require a concerted effort among physician stakeholders to affect changes within their billing regulations. For example, billing practices that allow patients in the transition years (18–30) with specific conditions to be seen as needed by the appropriate health care provider for changing symptoms without re-referral every six months will not only improve ease of access, but also decrease morbidity symptoms [6]. 

The complexity of navigating multisystem changes with complex and life-threatening conditions requires a specific kind of health care professional and health services. Until recently, there has been little focus on primary care providers in the transition process [55]. The role of a primary care home is now prominent in clinical guidelines on transition [29, 46]. Primary care philosophy recognizes that health transition must also include education, career choices, and independent living [46]. In pediatric care, nurse clinicians provide primary care management of complex youth and families in tertiary care settings. In adult services, there is no “safe passage” for young adults to an equivalent supportive care coordinator. Nursing experts understand the unique health, developmental, and social challenges of this population and are well suited to provide ongoing support for the coordination of future health care, as well as liaison for education and social services planning [54]. A joint report from the Canadian Health Research Foundation and Canadian Nurses Association (2012) has determined that community-based and nurse-led interdisciplinary teams that include a primary care physician are key components of effective and efficient provision of complex care to a complex group of patients. Multifaceted interventions that include both health and social interventions require specially trained or advanced practice nurses to supplement primary care physicians and other health care professionals. This coordinated model of care provides proactive and comprehensive community care to vulnerable populations with complex health conditions and social circumstances [56]. 

There is evidence of specialized resources for young adults with pedLTC in the United Kingdom where children's hospices have expanded their upper age limit into the young adult years, and new young adult specific hospices and hospice societies are being developed [1, 57]. Like pediatric hospice, young adult hospice (YAH) is philosophically different from adult hospice and can function as an episodic primary care home. The goals of YAH include anticipatory guidance about managing changing symptoms and planning for end of life through the entire trajectory of life, not just the last weeks and months. Young adult hospice homes and hospice societies can also encourage successful transition from pediatric to adult services, provide specialized symptom control, psychosocial, and spiritual resources, support independence, social networks, recreation, end of life decisions and care, and access to work and educational opportunities. Hospice services for family members can include respite and bereavement support [2, 54]. Young adults living in nonurban areas can be supported through telehealth symptom management, the expert support of a network of providers, and information about how to facilitate online social connections and navigate multisectoral services in their area. The input and voice of young adults in the development of hospice services are imperative to ensure that hospice services are responsive to their needs and life goals [1]. Engaging young adults with pedLTC in the development and ongoing support of hospice social networks and providing peer support about how to access health, education, vocational, and independent living resources will create meaningful work, volunteer, and social opportunities. 

## 5. Strengths and Limitations

While this is a small study and the results are not generalizable, it is among the first research studies to document transition experiences from pediatric to adult services from the perspective of young adults with pedLTC. Additionally, data was collected using an innovative method, the BBFG, to reach a medically fragile population. While there were instances of limited follow-through by some participants on the BBFG discussion threads, the resulting data was rich and creative. Further research opportunities include investigation of transition experiences for young adults with pedLTC from the perspectives of pediatric and adult care providers and family members. Developing collaborative training and sharing of resources between pediatric and adult providers and programs will improve the transition experience for young adults with pedLTC. 

## 6. Conclusions

After nearly a decade of effort toward implementing programs, protocols, and policies to improve transition from pediatric to adult care, very limited progress in new services has been made [3, 23, 29, 54]. Ideas and programs do not become diffused into normal practice without continual investment and appraisal of their status. For young adults with pedLTC, impasses continue to exist because of the history and culture of ingrained practice models, and deficient system policies and legislation for chronic illness transition [58]. These impasses are compounded by the complexity of their conditions, uncertain and sudden changes in their illness trajectory, lack of understanding by service providers of their conditions and resources required for living and educational and vocational pursuits, and lack of knowledgeable, supportive, and trusting relationships with adult care providers [59]. 

Much of the transition literature refers to the transition phase in terms of disempowerment “It feels like falling off a cliff” [17]. The loss of comprehensive services for young adults with pedLTC is profound and can be incredibly difficult without systems and family support. Despite these difficulties, all of the participants in this study were very optimistic about their future, exceeded the expectations provided by their health care providers, and anticipated that improving technologies and medications would continue to extend their lives. However, system barriers did limit and sometimes impede their efforts to live their young adult lives to their fullest, even while their health condition was stable. Resilience, community support, persistence, and hopefulness enabled these young adults to navigate and persevere through system barriers [60, 61]. 

Care coordination through YAH programs is a substantive addition for young adults with pedLTC, who are entering adult health care with complex lifelong histories, multiple medical interventions, and varying system navigation experiences [62]. It is imperative to implement multidisciplinary and multiagency system solutions focused on young adult priorities to guarantee that timely and seamless resources will continue to support these persons' health, educational, vocational, and social goals, in their quest to maximize their opportunities for young adult experiences in an abbreviated time frame. 

## 7. Notes

The terminology used to describe young adults with pediatric life-threatening conditions (pedLTC) varies among authors. Some choose to use pediatric life-limiting conditions (pedLLC) and others choose pediatric life-threatening diseases (pedLTD). I chose to use pedLTC to distinguish this population from (1) pedLTC which has been used to describe youth with conditions that may be life limiting, but with expectations to live well into their 1950s and 1960s, and from (2) pedLTD which does not reflect that many of these young adults do not have a specific disease, but rather complex conditions with multi system effect.

## Figures and Tables

**Table 1:** Propositions: up against the system.

(1) Youth with pedLTC are potentially juxtaposed between the hope of achieving new young adult milestones and their own end of life trajectory.	

(2) Health care transitions are embedded with the broader systems and are connected to family, social, educational, cultural, and religious factors [3, 23].	

(3) Development of comparable medical and support services did not follow interventions to increase life expectancy [3–5].	

(4) Current transitional services from integrated pediatric services to the adult health and social service sectors are limited in their breadth, scope, and effectiveness to meet the needs of young adults with pedLTC [16, 20]. This could result in increased morbidity, isolation, and inadequate support to fulfill their aspirations for intimacy, independence, education, work, and interpersonal relationships [6, 21].	

(5) Resiliency, vulnerability, personal factors (cognitive capacity, temperament, self-direction, imagination, values, meaning of transitional phase, and motives), parental influences (availability, history of acceptance to youth decision making, attitudes or preoccupation with youth's strengths or limits, and view of impact of youth's pedLTC on siblings and family), and external supports (parents, siblings, extended family, teachers, and community/religious/cultural mentors and volunteers) can influence the youth's experience of the transition process [21].	

(6) Health and other service providers in the adult care sector could have limited awareness of the complexity of the issues for youth with pedLTC and/or do not have a mandate and/or the resources to provide integrated services to young adults with pedLTC [30, 31, 33].	

**Table 2:** Participant characteristics for face-to-face interviews and bulletin board discussion groups *n* = 10.

Conditions	Number of participants	Age range and mean	Gender
Duchennes muscular dystrophy	4	20–28 23	Males = 4
Spinal muscular atrophy	3	21–26 23	Females = 3
Other: undiagnosed, brain tumor, Friedrichs Ataxia	3	20–22 21	Males = 2 Females = 1
